# Insulin-like growth factors in endometrioid adenocarcinoma: Correlation with clinico-pathological features and estrogen receptor expression

**DOI:** 10.1186/1471-2407-12-262

**Published:** 2012-06-21

**Authors:** Yuan-Jiao Liang, Qun Hao, Hui-Ming Zhang, Yuan-Zhe Wu, Jian-Dong Wang

**Affiliations:** 1 Department of Obstetrics & Gynecology, Jinling Hospital, Nanjing University School of Medicine, Nanjing, Jiangsu, 210002, China; 2 Department of Pathology, Jinling Hospital, Nanjing University School of Medicine, Nanjing, Jiangsu, China

**Keywords:** Endometrioid adenocarcinoma, Insulin-like growth factor, Estrogen receptor

## Abstract

**Background:**

Endometrial carcinoma is a common malignancy of female genital tract. Insulin-like growth factor is known to elicit estrogen-induced mitogenic activity and anti-apoptotic effect in endometrial tissues.

**Methods:**

The retrospective study investigated the expression of insulin-like growth factors, estrogen receptors and their associations in endometrioid adenocarcinoma (EAC) from 80 EAC patients in immunohistochemistry, and 58 EAC patients and 42 control patients in quantitative RT-PCR. The Pearson correlation analysis was used to analyze their correlations with clinic-pathological parameters.

**Results:**

Our results showed that insulin-like growth factor-1 and insulin-like growth factor-2 mRNA levels were higher in tumor tissues and tumor-adjacent tissues than those in control cells, and were inversely correlated with the malignancy of the tumor with a positive correlation with ERα and ERβ expression. Insulin-like growth factor-1R protein expression was correlated with clinical stage, and insulin-like growth factor-2R protein expression was inversely correlated with histological grade.

**Conclusions:**

Insulin-like growth factor system plays an important role in estrogen-induced endometrial carcinogenesis, and overexpression of insulin-like growth factor-1R in the advanced endometrioid adenocarcinoma is not estrogen-dependent.

## Background

Endometrial carcinoma is the most common malignancy of female genital tract in the United States [1]. Endometrioid adenocarcinoma (EAC) is the most common type of endometrial carcinoma, accounting for more than 80% of the cases [2]. EAC is known to be estrogen-related, and often occurs in postmenopausal women with a history of excessive exposure of estrogen. Unopposed estrogen can produce endometrial hyperplasia, and there is a strong association between endometrial hyperplasia and EAC [3], suggesting that EAC represents an estrogen-stimulated progression from endometrial hyperplasia to carcinoma. However, molecular mechanism underlying the association between estrogen and EAC has not been well established yet.

Estrogen exerts its biological effects through two distinct receptors, ERα and ERβ, which belong to the nuclear steroid superfamily. ERα is the predominant estrogen receptor in human uterus, and is required for endometrial proliferation in response to estrogen exposure [4]. ERα is believed to be a prognostic factor for the survival in endometrial cancers [5-7]. ERβ has been found in both the normal and malignant endometrium, and decreased expression of ERβ is associated with the malignancy of the endometrial cancers [8-10]. However, the association of ERα and ERβ expression with clinical features of endometrioid adenocarcinoma has not been well studied yet.

Insulin-like growth factor (IGF) is known to elicit estrogen-induced mitogenic activity and anti-apoptotic effect in endometrial tissues [11,12]. The IGF system includes two growth factors (IGF-1 and IGF-2), two receptors (IGF-1R and IGF-2R) and several binding proteins (insulin-like growth factor binding proteins, IGFBPs). Estrogens increase the expression of IGF-1 in the uterus, and IGF-1 is required to mediate their mitogenic effect on the edometrium [13,14]. In addition, IGF-1 and IGF-2 has been reported to be involved in the progression of endometrial adenocarcinoma [15]. The availability and biological activities of IGFs are controlled and modulated by IGFBPs. Progesterone has been reported to increase IGFBP synthesis to antagonize estrogen-induced cell proliferation [14,16]. Though accumulated evidence shows that IGF family peptides are linked with EAC, their exact roles in estrogen-induced EAC remain unclear.

This study aimed to investigate the mRNA and protein expression of IGF-1, IGF-2, IGF-1R, IGF-2R, and IGFBP-3 as well as ERα and ERβ, to analyze their correlation with surgico-pathological stage, histological grade and depth of myometrial invasion, and to study the relationship of IGF system with ERα and ERβ expression in the EAC. Our study identified that IGF system plays an important role in estrogen-induced endometrial carcinogenesis.

## Methods

### Patients and samples

The hysterectomy specimens from these patients were collected between January, 2007 and July, 2010. This study included tissue samples from 80 EAC patients for immunohistochemistry, and tissue samples from 58 EAC patients and 42 control patients for quantitative RT-PCR. The study was in compliance with the Helsinki Declaration, approved by review board, of Nanjing University School of Medicine and all subjects were gave their informed consent. The quantitative PCR used specimens from 58 patients with EAC and 42 control patients with no endometrial diseases. The median age of the patients with EAC was 61 years (range 33-79 years). The cancers were classified according to recommendations of International Federation of Gynecology and Obstetrics (FIGO) in 2009. The surgico-pathologic staging of the cancers was as follows: stage I (n = 33), stage II (n = 15), stage III (n = 10), and stage IV (n = 0). The pathologic grading of the cancers was as follows: G1 (n = 14), G2 (n = 31), and G3 (n = 13). The endometrium outside the cancer loci was selected as tumor adjacent group, including 24 cases with atypical hyperplasia, 4 cases with complex hyperplasia, and 3 cases with proliferative phase of endometrium. The tumor samples and their clear surgical margin samples were removed during surgery, and were examined by a pathologist. The tumor-adjacent samples were selected from the clear surgical margin, which was defined as the 1 cm^2^ areas of tissues outside the tumor loci without any neoplasmatic texture histologically. Each diagnosis was confirmed by pathological staining, and all patients did not undergo radiation therapy, chemotherapy and hormonal therapy. The control endometria were collected from 42 patients (median age, 58 years) who underwent hysterectomy, and were confirmed to be normal by pathologic examination. The control group included 24 cases with the proliferative phase of endometrium and 18 cases with the secretory phase of endometrium. Immunohistochemistry experiments used specimens from 80 patients with EAC. The median age of the patients with EAC was 65 years (range 33-84 years). The surgico-pathologic staging of the cancers was as follows: stage I (n = 45), stage II (n = 19), stage III (n = 16), and stage IV (n = 0). The pathologic grading of the cancers was as follows: G1 (n = 21), G2 (n = 45), and G3 (n = 14). The control group included 22 cases with normal endometrium, and 33 cases with atypical hyperplasia.

### RNA isolation and quantitative real-time RT-PCR

Total RNA was isolated from endometrial tissues from 58 patients with EAC and 42 control patients by using Trizol reagent (Invitrogen, USA) according to manufacturer’s protocol. RNA was reverse transcribed into complementary DNA using reverse transcription system. Quantitative real time RT-PCR was performed with primers and TaqMan probes listed in Table 1.

**Table 1:** The primers and TaqMan probes of IGF, ER genes used for RT-PCR

**Genes (Accession No.)**	**Primers and probe**	**size**	**Annealing temperature**
IGF-1 (NM_000618)	Forward: 5-AGCTGTGATCTAAGGAGGCTGG-3’	143 bp	57 °C
	Reverse: 5’-GCACTCCCTCTACTTGCGTTCTT-3’		
	Probe: 5’-(FAM)-TCAGCTCGCTCTGTCCGTGCCC-3’(TAMRA)		
IGF-1R (NM_000875)	Forward: 5’-CTTGTACATTCGCACCAATGCT-3’	83 bp	59 °C
	Reverse: 5’-CGATTAACTGAGAAGAGGAGTTCGA-3’		
	Probe: 5’-CTTCCATTCCCTTGGACGTTCTTTCAGC-3’		
IGF-2 (NM_000612)	Forward: 5’-AGGAGCTCGAGGCGTTCA-3’	65 bp	55 °C
	Reverse: 5’-GTCTTGGGTGGGTAGAGCATC-3’		
	Probe: 5’-AGGCCAAACGTCACCGTCCCC-3’		
IGF-2R (NM_000876)	Forward: 5’-GCGGCACACCCTATAACAATG-3’	74 bp	59 °C
	Reverse: 5’-CGCGTCTCGATCACAGAGAA-3’		
	Probe: 5’-AAGACACACACCGAGAGCTACGCTCATCA-3’		
IGFBP-3 (NM_000596)	Forward: 5’-CAGGAGACATCAGGAGAAGAAATTT-3’	117 bp	59 °C
	Reverse: 5’-TCCCGCCTCTCCATCCAT-3’		
	Probe: 5’-TTACCTGCCAAACTGCAACAAGAATGGATT-3’		
ERα (NM_000125)	Forward: 5’-AGAGGGCATGGTGGAGATCTT-3’	83 bp	59 °C
	Reverse: 5’-CAAACTCCTCTCCCTGCAGATT-3’		
	Probe: 5’-(FAM)TGATGACACTGCCACTCCTGAGGCA-3’(TAMRA)		
ERβ (NM_001437)	Forward: 5’-GACCACAAGCCCAAATGTGTT-3’	69 bp	59 °C
	Reverse: 5’-AACTGGCGATGGACCACTAAA-3’		
	Probe: 5’-(FAM)TGGCCAACACCTGGGCACCTTT-3’(TAMRA)		
GAPDH (NM_002046)	Forward: 5’-CCAGGTGGTCTCCTCTGACTT-3’	130 bp	59 °C
	Reverse: 5’-GTTGCTGTAGCCAAATTCGTTGT-3’		
	Probe: 5’-(FAM)AACAGCGACACCCACTCCTCCACC-3’(TAMRA)		

### Immunohistochemistry

Tissue sections (4 μM thick) were obtained from formalin-fixed and paraffin-embedded tissue blocks from 80 hysterectomy specimens. The tissue sections were immunohistochemically stained for IGF-1R, IGF-2R, ERα and ERβ, using EnVision method as previously described. Briefly, sections were washed in xylene to remove the paraffin, rehydrated with serial dilutions of alcohol, followed by a wash in PBS solution. The samples were then incubated in primary antibodies against IGF-1R (polyclonal rabbit anti-human IGF-1R, 1:50 dilution, Wuhan Boster*.* Bio-engineering Co*.,* Ltd*.,* Wuhan*,* China), IGF-2R (polyclonal rabbit anti-human IGF-2R, 1:50 dilution, Wuhan Boster*.* Bio-engineering Co*.,* Ltd*.,* Wuhan*,* China), ERα (monoclonal rabbit anti-human ERα, 1:50 dilution, Fujian Maixin. Biological Technolog*,* Fujian*,* China) and ERβ (monoclonal rabbit anti-human ERβ, 1:50 dilution, Fujian Maixin. Biological Technolog*,* Fujian*,* China) overnight at 4 °C. After the primary antibody was washed off, the components of the Envision-plus (DAKO) detection system were applied, and sections were counterstained with hematoxylin. The tissue sections from known breast cancer were used as positive control, and sections in which primary antibodies were omitted were used as negative control.

The immunostaining was examined under the light microscope by two observers blind to the experimental conditions, using an immunohistochemical scoring system according to the percentage of stained cells and the intensity of the immunoreactivity [17]. The agreement on the scores between the two observers was nearly 100%. In cases in which the observers disagreed in the score, the immunohistochemical scoring was repeated by both observers until the same score was achieved. The percentage of stained cells was scored as follows: 0 for no stained cells, 1 for <10% of stained cells, 2 for 10% to 50% of stained cells, and 3 for >50% of stained cells. The intensity of immunoreactivity was scored as follows: 0 for no staining, 1 for weak staining, 2 for moderate staining, and 3 for strong staining. The final immunoreactive score was determined by the sum of both the intensity score and the score for the percentage of positively stained cells. The negative, positive and strong positive immunoreactivity is defined by a final score of ≤ 1, 2-3, and ≥ 4, respectively.

### Statistical analysis

Statistical analyses were performed using SPSS 13.0. The values were presented as mean and standard deviation. If equal variances were achieved, one-way analysis of variance (ANOVA) was used for comparison of the difference in the means among cancer group, tumor-adjacent group and control group using LSD (least significant difference) method. If equal variances were not achieved, Welch approximate analysis of variance was performed to analyze the differences among groups using Tamhan’s T2 method. Categorical data were compared with chi square. The Pearson correlation analysis was applied to assess the relationship between IGFs and ERs and between IGFs and clinical pathological feature of EAC. Probability values less than 0.05 were considered statistically significant.

## Results

### Expression of IGFs and ERs in endometrioid adenocarcinoma at mRNA and protein levels

We tested the expression of IGF-1, IGF-1R, IGF-2, IGF-2R, IGFBP-3, ERα and ERβ mRNA on 58 tumor tissue samples and 31 tumor-adjacent endometrial tissue samples from patients with EAC and 42 normal endometrial tissue samples, using RT-PCR. The mRNA level of IGF-1, IGF-1R, IGF-2, IGF-2R, and IGFBP-3 was significantly higher in tumor and tumor-adjacent endometrium than that in control endometrium (p < 0.05, Table 2). The tumor endometrium expressed significantly lower levels of IGF-1, IGF-1R, and IGF-2, and significantly higher levels of IGF-2R and IGFBP-3, compared with tumor-adjacent endometrium (p < 0.05, Table 2). In addition, the mRNA level of ERα and ERβ was not significantly different between the tumor group and the control group (p > 0.05). The tumor-adjacent endometrium expressed higher levels of ERα and ERβ compared with both tumor endometrium and normal endometrium (p < 0.05, Table 2).

**Table 2:** The mRNA levels of IGF-1, IGF-1R, IGF-2, IGF-2R, IGFBP-3, ERα and ERβ in EAC, tumor-adjacent, and control groups

**groups**	**n**	**IGF-1**	**IGF-1R**	**IGF-2**	**IGF-2R**	**IGFBP-3**	**ERα**	**ERβ**
Control	42	0.58 ± 0.30	0.54 ± 0.32	0.33 ± 0.19	0.04 ± 0.03	4.58 ± 3.35	16.24 ± 10.10	50.10 ± 21.60
EAC	58	1.19 ± 00.79*	6.23 ± 3.98*	2.44 ± 2.32*	4.32 ± 2.98*	14.47 ± 12.31*	10.67 ± 7.63	31.44 ± 25.22
Tumor-adjacent	31	2.66 ± 01.73**#**	35.34 ± 22.02#	8.59 ± 7.13#	0.39 ± 0.36#	8.28 ± 4.57#	45.54 ± 33.58#	529.62 ± 269.7#

*p < 0.05 vs control,#*P* < 0.05 vs EAC (endometrioid adenocarcinoma) by one-way ANOVA.

**Figure 1:**
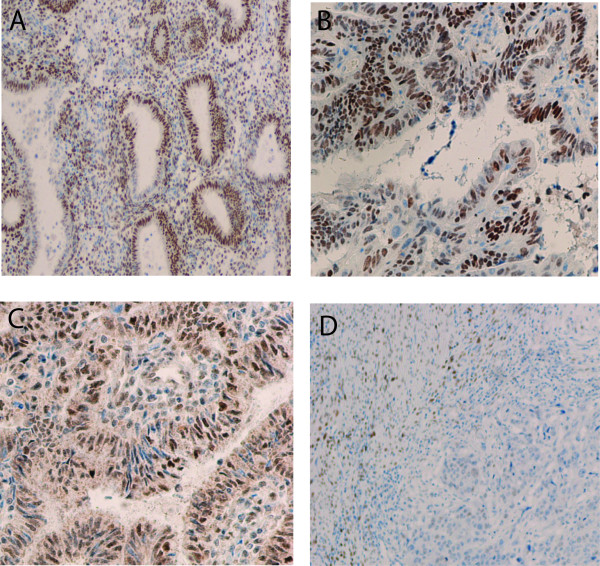
Expression of ERα using immunohistochemical analysis in normal endometrium (A) (×200), atypical hyperplasic endometrium (B) (×400), endometrial adenocarcinoma with high differentiation (C) (×200), and endometrial adenocarcinoma with low differentiation (D) (×100).

**Figure 2:**
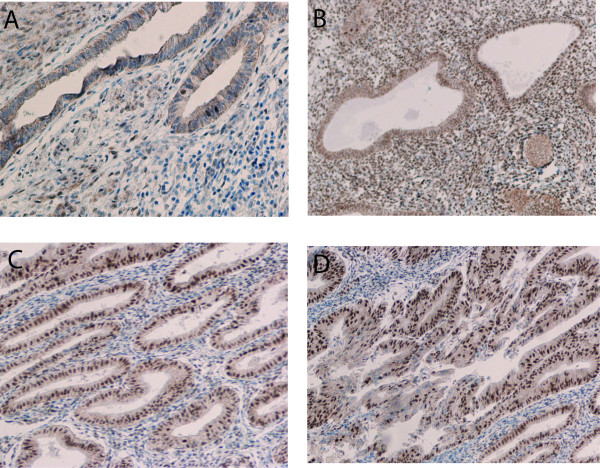
Expression of ERβ using immunohistochemical analysis in normal endometrium (A) (×200), atypical hyperplasic endometrium (B) (×200), endometrial adenocarcinoma with high differentiation (C) (×200), and endometrial adenocarcinoma with low differentiation (D) (×200).

**Figure 3:**
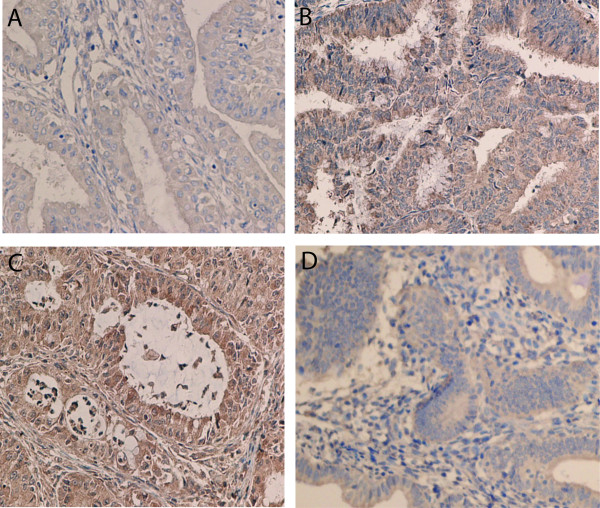
Expression of IGF-1R (A, B) and IGF-2R (C, D) using immunohistochemical analysis in endometrial adenocarcinoma with high differentiation (A) (×400), endometrial adenocarcinoma with low differentiation (B) (×400), endometrial adenocarcinoma with moderate differentiation (C) (×400), and endometrial adenocarcinoma with low differentiation (D) (×200).

We then detected the protein expression of IGF-1R, IGF-2R, ERα and ERβ on 80 tumor samples from patients with EAC, 33 samples from patients with atypical hyperplasia, 22 samples from control patients, using immunohistochemistry (Figure 1, 2, 3). Nuclear immunoreactivity was observed for both ERα and ERβ with no cytoplasmic immunoreactivity (Figure 1, 2). ERα immunoreactivity was strongly positive in normal endometrium and atypical hyperplasic endometrium with a positive staining rate ranging from 93.9% to 100%, while 73.8% of endometrioid adenocarcinoma exhibited the positive immunoreactivity to ERα with a strong positive staining rate of 12.5% (Table 3). In contrast, ERβ immunoreactivity was weakest in normal endometrium and the strongest in EAC. The strong positive staining rate was 13.6%, 25.5%, and 93.8% in normal endometrium, atypical hyperplasic endometrium and EAC, respectively (Table 3). For IGF-1R, the highest expression rate was seen in EAC, and the lowest expression rate was found in normal endometrium. The strong positive staining rates were 5%, 27.3%, and 40% in normal endometrium, atypical hyperplasic endometrium and EAC, respectively (Table 3). However, IGF-2R immunoreactivity occurred more frequently in atypical hyperplasic endometrium, and less frequently in EAC. The strong positive staining rates were 45.5%, 75.8%, and 12.5% in normal endometrium, atypical hyperplasic endometrium and EAC, respectively (Table 3).

**Table 3:** The immunostaining of IGF-1R, IGF-2R, ERα and ERβ in EAC, atypical hyperplasia, and control groups

**groups**	**n**	**ERα**		**ERβ**		**IGF-1R**		**IGF-2R**	
		**Positive (%)**	**Strong positive(%)**	**Positive (%)**	**Strong positive(%)**	**Positive (%)**	**Strong positive(%)**	**Positive (%)**	**Strong positive(%)**
Control	22	22(100)	22(100)	13(59.09)	3(13.64)	14(63.64)	1(5.0)	16(72.72)	10(45.45)
Atypical hyperplasia	33	32(95.65)	31(93.93)	26(78.79)	15(45.45)	25(75.76)	9(27.27)	27(81.82)	25(75.75)
EAC	80	59(73.75)	10(12.5)	78(97.5)	75(93.75)	72(90.0)	32(40.0)	15(18.75)	10(12.50)
*x*^2^		18.961	92.295	23.444	62.864	8.953	10.421	47.424	43.807
*P* value		0.0000	0.0000	0.0000	0.0000	0.011	0.005	0.0000	0.0000

### Association of IGF and ER expression with clinico-pathological features of EAC

We investigated the association of IGF-1, IGF-1R, IGF-2, IGF-2R, IGFBP-3, ERα and ERβ mRNA expression with surgico-pathological stage in EAC (Table 4). Out of 58 cases evaluated, there were 33 stage I, 15 stage II, and 10 stage III tumors. The expression of IGF-1, IGF-2 and IGF-2R was decreased with the increased stage of tumors (P < 0.05), though no significant difference was found between stage II and stage III tumors in IGF-2 expression, and between stage I and stage II tumors in IGF-2R expression. There was no significant change in IGF-1R and IGFBP-3 expression with the stage of the tumors. Decreased expression of ERα and ERβ was found with the increased stage of tumors (P < 0.05).

**Table 4:** The relationship between the IGF and ER mRNA expression and clinico-pathological features of endometrioid adenocarcinoma

**Clinical pathology (n)**		**IGF-1**	**IGF-2**	**IGF-1R**	**IGF-2R**	**IGFBP-3**	**ERα**	**ERβ**
Surgico-pathological stage	I(33)	1.70 ± 0.73▾	3.28 ± 2.17▾	1.62 ± 0.86	4.97 ± 2.77	14.91 ± 13.03	16.57 ± 4.1▾	42.04 ± 23.28
	II(15)	0.58 ± 0.09*	1.73 ± 2.47	1.84 ± 0.96	4.70 ± 2.96*	13.25 ± 13.32	4.33 ± 2.39*	25.70 ± 24.47*
	III(10)	0.44 ± 0.02**#**	0.72 ± 1.31**#**	1.50 ± 0.79	1.60 ± 2.50**#**	14.83 ± 16.79	0.72 ± 0.33**#**	5.07 ± 2.11**#**
Histological grade	G1(14)	1.44 ± 0.24	3.25 ± 2.27	1.53 ± 1.92	4.75 ± 3.01	16.22 ± 14.47	16.85 ± 4.37▴	41.93 ± 26.78
	G2(31)	1.39 ± 0.95**$**	2.53 ± 2.38**$**	1.32 ± 0.99	4.92 ± 2.63**$**	11.78 ± 11.27	11.70 ± 6.94**$**	35.08 ± 23.96**$**
	G2(13)	0.47 ± 0.07**&**	1.34 ± 2.02**&**	1.66 ± 0.84	2.42 ± 3.27**&**	18.99 ± 16.87	1.55 ± 1.79**&**	11.47 ± 16.42**&**
Depth of myometrial invasion	≤ 50%(39)	1.47 ± 0.79	3.14 ± 2.27	1.55 ± 0.90	5.26 ± 2.68	15.82 ± 12.78	14.28 ± 5.64	41.48 ± 24.10
	>50%(19)	0.63 ± 0.46**@**	0.98 ± 1.76**@**	1.15 ± 0.78	2.39 ± 2.75**@**	11.69 ± 15.01	3.26 ± 5.86**@**	10.83 ± 12.55**@**

▾p < 0.05 stage I vs stage II;*P < 0.05 stage II vs stage III; #*P* < 0.05 stage I vs stage III;▴*P* < 0.05 G1 vs G2;$*P* < 0.05 G2 vs G3;&*P* < 0.05 G1 vs G3;@*P* < 0.05 vs ≤50% of mymetrial invasion by one-way ANOVA.

We examined the association of IGF-1, IGF-1R, IGF-2, IGF-2R, IGFBP-3, ERα and ERβ mRNA expression with histological grade in EAC (Table 4). The histological grade of the 58 cases was classified into 3 groups: well differentiated (G1, n = 14), moderate-differentiated (G2, n = 31) and poor-differentiated (G3, n = 13). The mRNA expression of IGF-1, IGF-2, and IGF-2R was decreased with the lack of differentiation with a significant difference between G1 and G3 groups and between G2 and G3 groups. No significant change was identified in the mRNA expression of IGF-1R and IGFBP-3 among G1, G2 and G3 groups. The mRNA expression of ERα and ERβ was decreased with the lack of differentiation with a significant difference among G1, G2 and G3 groups.

We also tested the association of IGF-1, IGF-1R, IGF-2, IGF-2R, IGFBP-3, ERα and ERβ mRNA expression with the depth of myometrial invasion in EAC (Table 4). Of the 58 cases, superficial myometrial invasion (≤ 50% of myometrium) was present in 39 cases, and deep myomytrial invasion (> 50% of myometrium) was present in 19 cases. The mRNA expression of IGF-1, IGF-2 and IGF-2R, but not IGF-1R and IGFBP-3, was decreased with the increased depth of myometrial invasion. The ERα and ERβ mRNA expression was significantly lower in the EAC with deep myometrial invasion than that with superficial myometrial invasion.

We investigated the association of IGF-1R, IGF-2R, ERα and ERβ protein expression with the surgico-pathological stage, histological grade, and depth of myometrial invasion in the EAC (Table 5). The percentage of IGF-1R-positive staining was increased, and the percentage of IGF-2R-positive staining was decreased with the increased surgico-pathological stage, histological grade, and depth of myometrial invasion. In addition, decreased ERα-positive immunostaining was observed with the increased surgico-pathological stage, histological grade, and depth of myometrial invasion. However, though strong positive staining for ERβ in 93.75% of EAC samples, ERβ-positive immunostaining exhibited no significant difference in the surgico-pathological stage, histological grade, and depth of myometrial invasion.

**Table 5:** The relationship between the IGF-1R, IGF2R, ERα and ERβ protein expression and clinico-pathological features of endometrioid adenocarcinoma

**Clinical pathology**		**n**	**ERα**		**ERβ**		**IGF-1R**		**IGF-2R**	
			**Positive (%)**	**Strong positive(%)**	**Positive (%)**	**Strong positive(%)**	**Positive (%)**	**Strong positive(%)**	**Positive (%)**	**Strong positive(%)**
Surgico-pathological stage	I	45	40(88.9)	19(42.2)	45(100)	43(95.6)	39(86.7)	7(15.6)	11(24.4)	5(11.1)
	II	19	14(73.7)	0(0)	18(94.7)	13(68.4)	17(89.5)	10(52.6)	2(10.5)	0(0)
	III	16	5(31.3)	0(0)	15(93.8)	16(100)	16(100)	15(93.8)	2(12.5)	0(0)
*x*^2^			18.935	7.334	3.388	3.225	3.886	31.727	2.316	2.544
*P* value			0.000	0.013	0.184	0.237	0.143	0.000	0.314	0.291
Histological grade	G1	21	21(100)	9(42.8)	21(100)	21(100)	13(61.9)	4(19.1)	10(47.6)	5(23.8)
	G2	45	33(73.3)	10(22.2)	45(100)	38(84.4)	45(100)	15(33.3)	4(19.1)	0(0)
	G3	14	5(35.7)	0(0)	13(92.9)	13(92.9)	14(100)	13(92.9)	1(7.1)	0(0)
*x*^2^			21.663	2.543	3.547	3.844	24.103	20.972	13.974	2.689
*P* value			0.000	0.447	0.170	0.163	0.000	0.000	0.001	0.304
Depth of myometrial invasion	≤1/2	58	53(91.4)	13(22.47)	56(96.6)	51(87.9)	50(86.2)	14(24.1)	11(19.0)	5(8.6)
	>1/2	22	6(27.3)	6(27.3)	22(100)	21(95.5)	22(100)	18(81.8)	4(18.2)	0(0)
*x*^2^			33.853	3.345	0.006	0.102	2.013	22.111	0.000	1.015
*P* value			0.000	0.264	0.936	0.845	0.156	0.000	1.000	0.933

### Correlation analysis of IGF and ER expression with clinico-pathological features of EAC

A strong inverse correlation of IGF-1 and IGF-2 mRNA expression, a weak inverse correlation of IGF-2R mRNA expression, and no correlation of IGF-1R and IGFBP-3 mRNA expression were found with the surgico-pathological stage, histological grade, and depth of myometrial invasion (Table 6). Similar to IGF-1 and IGF-2, a strong inverse correlation of ERα and ERβ was observed with the surgico-pathological stage, histological grade, and depth of myometrial invasion (Table 6).

**Table 6:** Correlation analysis of IGF and ER mRNA expression with clinico-pathological features of EAC

	**Surgico-pathological stage**	**Histological grade**	**Depth of myometrial invasion**
IGF-1	-0.8909*	-0.6434*	-0.6704*
IGF-2	-0.5867*	-0.3907*	-0.5826*
IGF-1R	-0.0901	0.1587	0.0675
IGF-2R	-0.3519*	-0.2842*	-0.4663*
IGFBP-3	-0.0549	0.1012	-0.1920
ERα	-0.8807*	-0.6906*	-0.6803*
ERβ	-0.6384*	-0.4958*	-0.6616*

*P < 0.05 using Pearson correlation analysis with linear regression.

IGF-1R protein expression was correlated with the surgico-pathological stage, histological grade, and depth of myometrial invasion, and IGF-2R protein expression was inversely correlated with histological grade, but not with the surgico-pathological stage and depth of myometrial invasion (Table 7). In addition, ERα protein expression was inversely correlated with surgico-pathological stage and histological grade, but ERβ protein expression exhibited no correlation with all the studied parameters (Table 7).

**Table 7:** Correlation analysis of IGF-1R, IGF-2R, and ERα and ERβ protein expression with clinico-pathological features of EAC

	**Surgico-pathological stage**	**Histological grade**	**Depth of myometrial invasion**
ERα	-O.468*	-0.386*	-0.238*
ERβ	0.321	0.209	0.096
IGF-1R	0.616*	0.628*	0.546*
IGF-2R	-0.134	-0.379*	-0.104

*P < 0.05 using Pearson correlation analysis with linear regression.

### Correlation of IGF mRNA expression with ERα and ERβ expression

We further study the correlation of IGF-1, IGF-1R, IGF-2, IGF-2R, and IGFBP-3 mRNA expression with ERα and ERβ mRNA expression in tumor, tumor-adjacent and control endometria (Table 8). In the control group, there was no correlation of IGF-1, IGF-1R, IGF-2, IGF-2R, and IGFBP-3 expression with ERα and ERβ expression. In the tumor group, IGF-1 and IGF-2 levels were strongly correlated with ERα expression (r = 0.6439 for IGF-1, r = 0.5228 for IGF-2), and were weakly correlated with ERβ expression (r = 0.4155 for IGF-1, r = 0.3555 for IGF-2). IGF-2R expression was weakly correlated with both ERα (r = 0.2970) and ERβ (r = 0.2756) expression. No correlations of either ERα or ERβ expression were found with IGF-1R and IGFBP-3 expressions. In tumor-adjacent group, the IGF-2 level was strongly correlated with both ERα (r = 0.8502) and ERβ (r = 0.9327) expressions. Strong correlation of IGF-1R expression was observed with ERα expression (r = 0.5545), but not with ERβ expression (r = -0.0399). There were no correlations of IGF-1, IGF-2R and IGFBP-3 with either ERα or ERβ expressions.

**Table 8:** The analysis of the correlation between the mRNA expression of IGFs with ER subtypes in three groups

**groups**		**IGF-1**	**IGF-1R**	**IGF-2**	**IGF-2R**	**IGFBP-3**
control	ERα	0.2151	-0.1216	-0.1543	0.1938	-0.1150
	ERβ	0.1881	0.0719	-0.1295	-0.1145	-0.2147
EAC	ERα	0.6439*	0.2537	0.5228*	0.2970*	0.1519
	ERβ	0.4155*	0.1429	0.3555*	0.2756*	0.0583
Tumor-adjeacent	ERα	0.3009	0.5545*	0.8502*	-0.0699	-0.2465
	ERβ	0.0925	-0.0399	0.9327*	0.2615	-0.2710

*P < 0.05 using Pearson correlation analysis with linear regression.

## Discussion

IGF family including IGF-1, IGF-2, their receptor IGF-1R and IGF-2R, and IGFBPs, regulates cell proliferation, metabolism and differentiation in normal cells, and plays an important role in growth and progression of many tumors [18]. In endometrium, IGF family is believed to function as a mediator of estrogen actions through paracrine/autocrine mechanisms, and is associated with estrogen-induced endometrial carcinogenesis [11-15]. However, the function of IGF system in the EAC remains unclear. Here we investigate the expression of IGF family using RT-PCR and immunohistochemistry, and analyze its correlations with clinicopathological features and ER expression in the EAC. We find that expression of IGF-1, IGF-2 and IGF-2R mRNA is inversely correlated with the surgico-pathological stage, histological grade, and depth of myometrial invasion, and is correlated with ERα and ERβ expression in EAC. IGF-1R protein expression is correlated with surgico-pathological stage, histological grade, and depth of myometrial invasion, and IGF-2R protein expression is inversely correlated with histological grade, but not with the surgico-pathological stage and depth of myometrial invasion. In addition, we also find that the expression of IGF-1, IGF-2, and IGF-2R, but not IGF-1R, is correlated with ERα and ERβ expression. Since ERα and ERβ expression is associated with EAC carcinogenesis, our results suggest that IGF-1, IGF-2 and IGF-2R may play an important role in estrogen-induced endometrial carcinogenesis, and overexpression of IGF-1R in the EAC is not estrogen-dependent.

Estrogens, acting through ERα and ERβ, play a key role in development and progression of EAC. High levels of ERα are believed to be favorable for prognosis and treatment of EAC [5-7]. Consistent with this idea, we find that the normal endometrium expresses higher levels of ERα than the EAC, and decreased expression of ERα is correlated with the malignancy of the tumor. However, the expression of ERβ protein exhibits no correlation with the malignancy of EAC. Though excessive estrogen has been associated with the carcinogenesis of EAC, the mechanisms underlying the estrogen-mediated carcinogenesis remain unclear. Growing evidence shows the involvement of IGF system in the carcinogenesis and progression of EAC. Increased levels of plasma IGF-1 in women has been associated with the increased risk of EAC [19,20]. IGF-1 and IGF-2 has been reported to be involved in the progression of EAC [15]. The involvement of both estrogen and IGF signaling pathway in the EAC suggests that the interaction of estrogen with IGF signaling pathways may be important in the pathogenesis of EAC similar to that in the breast cancer [21]. This idea is supported by the reports that estrogen stimulates the expression of IGF-1, and IGF-1 is required to mediate estrogen action in the endometrium [13,14,22].

In our study, the IGF-1 and IGF-2 mRNA levels are much higher in tumor cells and tumor-adjacent cells than those in control tissues, suggesting that IGF-1 and IGF-2 may be a critical mediator for transforming from normal cell into tumor cells. This finding agrees with reports that IGF-1 and IGF-2 elicit tumorigenesis through autocrine mechanism [11,12]. However, a strong inverse correlation of IGF-1 and IGF-2 expression is found with the surgico-pathological stage, histological grade, and depth of myometrial invasion of EAC, suggesting that IGF-1 and IGF-2 function through autocrine mechanism may not be critical in the development of the malignancy of EAC. Our study can not exclude the possibility that IGF-1 and IGF-2 play a critical role in the development of EAC, since we did not test the plasma levels of IGFs which have been associated with EAC [19,20]. In addition, the tumor-adjacent endometrium, including mostly atypical hyperplasia, expresses higher level of IGF-1 and IGF-2 mRNA than EAC, further suggesting that IGF-1 and IGF-2 may play a role in the transformation to atypical hyperplasia, but the transformation from atypical hyperplasia into tumor may be IGF-independent. This idea is supported by the report that melanoma is sensitive to IGF-1 at the early stage, but insensitive to IGF-1 at the late stage of development [23]. In addition, the finding that IGF-1 and IGF-2 expression is correlated with the expression of both estrogen receptors is consistent with the idea that IGF expression is regulated by estrogen, and IGF may mediate estrogen-induced cell proliferation in the endometrium [13].

Effects of IGF-1 and IGF-2 are mediated through two receptors: IGF-1R and IGF-2R. Both IGF-1 and IGF-2 interact with IGF-1R, and IGF-2 also acts on IGF-2R. IGF-1R is regarded as a mediator for cancer cell proliferation, differentiation, growth and progression [18,24]. In agreement with the reports that overexpression of IGF-1R occurs in a variety of human cancer [24,25], we identify that IGF-1R protein expression is highest in the EAC, and is correlated with the surgico-pathological stage, histological grade, and depth of myometrial invasion of EAC. The percentage of IGF-1R-possitive cells is increased with the increased surgico-pathological stage, histological grade, and depth of myometrial invasion. However, since ERα protein expression is inversely correlated with the malignancy of EAC, no correlation of IGF-1R expression is observed with either ERα or ERβ expression, suggesting that overexpression of IGF-1R in the advanced EAC is not estrogen-dependent. In contrast, IGF-2R, structurally and functionally different from IGF-1R, is expressed at a low level in the EAC, and is reversely correlated with the malignancy of the EAC. This agrees with the fact that IGF-2R functions as a tumor suppressor to sequester IGF-2 from circulation [26,27]. In addition, the IGF-2R expression was significantly correlated with both ERα and ERβ expression in the EAC, suggesting that IGF-2R expression is estrogen-dependent.

The molecular mechanisms of estrogen-induced expression of IGF1, IGF2 and IGF-1R and IGF-2R are largely unknown. It has been reported that estrogen treatment increases IGF1 mRNA expression in EAC cells [28], possibly through regulation of IGF-1 gene promoter and a transcription factor C/EBPδ [29]. It is likely that estrogen can induce IGF-1, IGF-2, and their receptors through modulating their gene promoters and transcription factors. In this study, we find that the expression of IGF-1, IGF-2, and IGF-2R, is correlated with ERα and ERβ expression, suggesting that the expression of IGF-1 and IGF-2 and IGF-2R may be regulated by estrogen, and thus may play an important role in estrogen-induced endometrial carcinogenesis. Overexpression of IGF-1R in the EAC is not correlated with ERα and ERβ expression in the EAC, suggesting that regulation of IGF-1R may be not estrogen-dependent. The mechanism underlying IGF-1R expression is unclear. However, it is reported that overexpression of IGF-1R in a mouse model of transgenic expression of a constitutively active IGF-1R induces tumor development without the stimulation by any exogenous IGF-1R agonists or estrogen. An estrogen-independent mechanism underlying the overexpression of IGF-1R identified in this study remains to be further investigated.

The biological function of IGF peptides are also modulated by a family of IGF binding proteins, which are found in the circulation and in the extracellular fluids. IGFBP-3 is the predominant IGFBP in the plasma, and IGFBP-3 has been associated with endometrial carcinoma in postmenopausal women [30]. In addition, genetic variants in IGFBP-3, not IGFBP-1 is associated with the risk of endometrial carcinoma [31]. However, the expression of IGFBP-3 in the EAC and its association with the malignancy of the cancer and the expression of estrogen receptors remain unclear. Our study shows that no correlation of IGFBPs expression is found with the malignancy of EAC and the expression of estrogen receptors, suggesting that the IGFBP-3 is not critical in the development of EAC, and IGFBP-3 expression in the EAC is not estrogen-mediated.

There are some limitations to this study. First, this study included tissue samples from 80 EAC patients, but we only included tissue samples 58 EAC patients for quantitative RT-PCR. These samples used for RT-PCR were examined by a pathologist after the tumor was removed during surgery, and those samples without the pathologist’s examination were excluded. Though the sample size is large enough to compare the difference in the mRNA expression between the EAC and control, a large size of samples may be better to fully examine the mRNA expression of IGFs in the EAC. Second, our control samples included endometrial tissues in the proliferative phase and in the secretory phase. Due to the limited samples in the control study, we only tested the 24 control tissues in the proliferative phase and the 18 control tissues in the secretory phase. We do not find any significant difference in IGFs expression between tissues in the proliferative phase and in the secretory phase (data not shown), though the gene expression of IGFs in the proliferative and the secretary phase may be changed. A significant difference in the expression of IGFs between the control and the EAC is found in our study, suggesting that the sample size and the control samples with different endometrial phases do not affect our results greatly. Third, we did not test the plasma levels of IGFs which are reported to be associated with EAC. The expression of IGFs in the tumor reflects the synthesis and release of IGFs from the tumor, and the plasma levels of IGFs are affected by the synthesis and release of IGFs from many organs, especially the liver. It is worthy to investigate the correlation of plasma IGFs with the surgico-pathological stage, histological grade, and depth of myometrial invasion of EAC in the future.

## Conclusion

The present study has indicated that insulin-like growth factor system plays an important role in estrogen-induced endometrial carcinogenesis, and overexpression of insulin-like growth factor-1R in the advanced endometrioid adenocarcinoma is not estrogen-dependent.

## Abbreviations

IGF: Insulin-like growth factors; ER: Estrogen receptors; EAC: Endometrioid adenocarcinoma; IGFBP: Insulin-like growth factor binding proteins.

## Competing interests

The authors declare that they have no competing interests.

## Authors' contributions

YJL carried out the molecular studies, and drafted the manuscript. QH carried out the immunoassays. HMZ participated in the design of the study and performed the statistical analysis. YZW and JDW conceived of the study, and participated in its design and coordination and helped to draft the manuscript. All authors read and approved the final manuscript.

## Pre-publication history

The pre-publication history for this paper can be accessed here:

http://www.biomedcentral.com/1471-2407/12/262/prepub
